# Characterizing hip joint morphology using a multitask deep learning model

**DOI:** 10.1093/jhps/hnae041

**Published:** 2024-12-12

**Authors:** Bardia Khosravi, Lainey G Bukowiec, John P Mickley, Jacob F Oeding, Pouria Rouzrokh, Bradley J Erickson, Rafael J Sierra, Michael J Taunton, Emmanouil Grigoriou, Cody C Wyles

**Affiliations:** Department of Orthopedic Surgery, Mayo Clinic, 200 First Street SW, Rochester, MN 55905, USA; Department of Radiology, Mayo Clinic, 200 First Street SW, Rochester, MN 55905, USA; Department of Orthopedic Surgery, Mayo Clinic, 200 First Street SW, Rochester, MN 55905, USA; Department of Orthopedic Surgery, Mayo Clinic, 200 First Street SW, Rochester, MN 55905, USA; Department of Orthopedic Surgery, Mayo Clinic, 200 First Street SW, Rochester, MN 55905, USA; Department of Orthopedic Surgery, Mayo Clinic, 200 First Street SW, Rochester, MN 55905, USA; Department of Radiology, Mayo Clinic, 200 First Street SW, Rochester, MN 55905, USA; Department of Radiology, Mayo Clinic, 200 First Street SW, Rochester, MN 55905, USA; Department of Orthopedic Surgery, Mayo Clinic, 200 First Street SW, Rochester, MN 55905, USA; Department of Orthopedic Surgery, Mayo Clinic, 200 First Street SW, Rochester, MN 55905, USA; Department of Orthopedic Surgery, Mayo Clinic, 200 First Street SW, Rochester, MN 55905, USA; Department of Orthopedic Surgery, Mayo Clinic, 200 First Street SW, Rochester, MN 55905, USA; Department of Clinical Anatomy, Mayo Clinic, 200 First Street SW, Rochester, MN 55905, USA

## Abstract

Deep learning is revolutionizing medical imaging analysis by enabling the classification of various pathoanatomical conditions at scale. Unfortunately, there have been a limited number of accurate and efficient machine learning (ML) algorithms that have been developed for the diagnostic workup of morphological hip pathologies, including developmental dysplasia of the hip and femoroacetabular impingement. The current study reports on the performance of a novel ML model with YOLOv5 and ConvNeXt-Tiny architecture in predicting the morphological features of these conditions, including cam deformity, ischial spine sign, dysplastic appearance, and other abnormalities. The model achieved 78.0% accuracy for detecting cam deformity, 87.2% for ischial spine sign, 76.6% for dysplasia, and 71.6% for all abnormalities combined. The model achieved an Area under the Receiver Operating Curve of 0.89 for ischial spine sign, 0.80 for cam deformity, 0.80 for dysplasia, and 0.81 for all abnormalities combined. Inter-rater agreement among surgeons, assessed using Gwet’s AC1, was substantial for dysplasia (0.83) and all abnormalities (0.88), and moderate for ischial spine sign (0.75) and cam deformity (0.61).

## Introduction

Abnormal hip morphology is an important risk factor for hip pain, functional decline, and osteoarthritis (OA) development [1]. Thorough evaluation of radiographic properties of the hip joint is essential for diagnosis, treatment planning, and lifelong prognostication. Computer-assisted diagnosis has recently advanced through improvements in image processing, computational power, computer vision and machine learning techniques [2]. It has immense potential in improving the recognition of morphological hip derangements, particularly if deep learning is employed. Advances in ML have gained traction as they continue to excel in medical imaging recognition tasks [3–6]. However, a limited number of these technologies have been applied for computer vision analysis of radiographs displaying femoroacetabular impingement (FAI) and developmental dysplasia of the hip (DDH) [7–11].

Radiography, along with clinical examination, has traditionally been used to diagnose morphological pathologies including FAI and DDH. Anteroposterior (AP) pelvic radiographs serve as the initial imaging workup in individuals >6 months [12]. Development and ossification of the femur and acetabulum can be assessed in addition to pertinent radiographic features including center-edge angle, acetabular index, head–neck offset ratio, relationship to Hilgenreiner’s, Perkin’s and Shenton’s lines, and ischial spine sign.

FAI can occur secondary to three distinct phenotypes: cam morphology, pincer morphology, and mixed cam and pincer morphology. Cam deformity is represented by asphericity of the femoral head. Pincer morphology, involving over-coverage of the anterosuperior acetabulum, may be identified by observing a center-edge angle >40°, acetabular index <0°, or the presence of crossover or posterior wall signs. A positive ischial spine sign, representing the protrusion of the ischial spine medial to the iliopectineal line on standard AP radiograph, also indicates acetabular retroversion and is seen with pincer morphology. In both cam and pincer morphology, abnormal morphology of the hip joint causes pain and functional impairment typically in adolescents and young adults.

DDH, a disorder caused by abnormal development secondary to capsular laxity and mechanical instability, can cause radiographic findings ranging from dysplasia to hip dislocation. DDH is typically characterized by anterior or lateral center-edge angles <20°, Tonnis angles >10°, and acetabular index <0°.

Although diagnosing and characterizing hip morphological abnormalities increasingly rely on 3D imaging modalities, plain radiographs remain the gold standard for screening and initial workup. Expert classification of these abnormalities has shown poor inter- and intra-rater reliability in prior work [13]. Thus, the purpose of this study was to evaluate the ability of DL models to classify basic hip morphological abnormalities compared to human expert evaluation. The authors hypothesize that performance on such a task would be moderate given the difficulty in establishing a consensus human-driven ground truth in a dataset, which would be important to establish as DL models become more prevalent in hip preservation investigations and clinical care.

## Materials and methods

### Dataset description

The study received institutional review board approval. A total of 500 patients <50 years old at the time of their total hip arthroplasty (THA) were retrospectively reviewed. Cases were selected from an extensive institutional registry including all joint replacement operations since 1969 [14]. Patient radiographs from 2001 to the present were included. The dataset was partitioned at the patient level into a 4:1 ratio, resulting in 400 cases allocated to the training and tuning set and 100 cases to the final test set.

An orthopedic surgery resident and a medical student with 2 years of orthopedic surgery research experience (J.M., J.O.) annotated the training and testing datasets. They underwent three 1-h training sessions with two fellowship-trained, board-certified hip surgeons (C.W., E.G.). Case annotations with discrepancies were adjudicated by the supervising surgeons. The annotations identified four specific characteristics while considering the laterality of each: (i) ischial spine sign; (ii) femoral head cam deformity; (iii) hip dysplasia; and (iv) any abnormalities, encompassing the previous three categories along with other potential morphological irregularities. Annotations were determined at the discretion of each evaluator, without instruction to attend to a specific angle or metric in order for the model to mimic the classification process of a human evaluator. The testing dataset was annotated by an orthopedic surgery resident and a fellowship-trained, board-certified hip surgeon (L.B., E.G.).

### Joint localization

A previously validated object detection model employing YOLOv5 architecture was utilized to localize regions of interest (ROIs) within AP pelvic radiographs [15]. It was designed to identify laterality and presence of orthopedic hardware [16].

The model was trained on a curated dataset of labeled AP radiographs. Each image in the dataset was annotated with bounding boxes around the joint area, considering critical anatomical landmarks, including the greater and lesser trochanters, the pubic symphysis, and the acetabular sourcil to ensure precise localization. Upon training, the model outputs four coordinates that define a bounding box encompassing the joint and specifies laterality and presence of hardware. Boundaries of the box align with the bottom of the lesser trochanter inferiorly, the top of the greater trochanter superiorly, the pubic symphysis medially, and the lateral border of the greater trochanter laterally.

Joint ROIs that contained hardware were removed. In AP pelvic radiographs in which hardware was present in one joint, that joint was excluded from analysis and the contralateral joint, if free of hardware, was included in the analysis. This approach ensured a focus on native joint morphology.

### Morphology characterization

A convolution-based DL model, ConvNeXt-Tiny, was used to analyze hip morphology. It was designed to predict ischial spine sign, dysplasia, cam deformity, other abnormalities, and patient sex. Incorporating the auxiliary prediction of patient sex stabilized the model’s training—a strategy previously shown to enhance learning efficiency in complex models [17]. Due to the limited number of training samples, 10-fold cross-validation was employed. A total of 400 training-tuning cases were employed in this phase. An ensemble technique was used for the final assessment to combine predictions from all 10 models to average their outputs, mitigate variance, and boost overall prediction accuracy.

Image augmentation techniques, including horizontal flipping, rotation (−45 to +45 degrees), scaling (0.9–1.1×), and translation (up to 15 pixels in any direction), were employed during model training to artificially increase the diversity of the dataset and make the model more robust to variations in new, unseen images. The model processed images at a resolution of 384 pixels squares, with a batch size of 16 and a learning rate of 0.00001. The Lion optimizer was used for faster convergence [18]. Exponential moving average (EMA) of the weights was used with a decay factor of 0.9999. This method, coupled with a weight decay of 0.00004 helped prevent overfitting and resulted in a more generalizable model.

Binary cross-entropy with label smoothing (set to 0.1) was chosen as a loss function to allow each characteristic to be predicted as a binary outcome (present or absent), while label smoothing helped prevent the model from becoming overly confident in its predictions. The model was trained to a maximum of 500 epochs, and the best-performing model was selected based on the lowest validation loss value. All models were developed using the PyTorch library (v2.0.0) and trained on four A100 NVIDIA (Santa Clara, CA, USA) graphical processing units (GPUs).

### Evaluation

To assess the DL model’s performance, AUROC, F1-score, accuracy, sensitivity, specificity, positive and negative predictive values, thresholded based on Youden’s index, were reported. A fellowship-trained hip preservation surgeon (E.G.) established the final ground truth for the test set.

Inter-rater reliability was calculated among all the annotators (E.G. J.M., J.O., L.B.). This multitiered approach determined the consistency across annotations from individuals with different clinical experience levels. The inter-reader agreement was calculated using Gwet’s AC1. Values >0.6 on Gwet’s AC1 scale are indicative of high agreement, reflecting a reliable consensus among the annotators regarding the presence of hip joint characteristics and the prediction of the patient’s sex. Statistical significance was determined with a *P*-value threshold of <.05.

## Results

Images from 500 patients (mean age 47 years, 49% female) were analyzed. The ensemble pipeline processes each radiograph (including the pre-processing) in 832 ms (averaged over the test set).

The trained model achieved a mean average precision (mAP) of 99.4%, reflecting its reliability and accuracy in automatically detecting and localizing hip joints on radiographs. This high performance enables the application of this model for automatic data preprocessing, facilitating detailed and accurate assessment of joint conditions.

Table 1 presents key performance metrics of the model. The model achieved 87.2% accuracy for detecting ischial spine sign, 78.0% accuracy for cam deformity, 76.6% for dysplasia, and 71.6% for all abnormalities. The model demonstrated high specificity for ischial spine sign (96.0%) and all abnormalities (85.3%). Sensitivity was high for cam deformity (80.0%) and dysplasia (75.0%). The positive predictive value (PPV) was high for all abnormalities (93.5%) and ischial spine sign (87.5%).

**Table 1.** Performance metrics of the DL model for detecting hip joint abnormalities.

Abnormality (%)	Accuracy (%)	Sensitivity (%)	Specificity (%)	PPV (%)	NPV (%)	F1-score	AUROC
Ischial spine sign	87.2	66.7	96.0	87.5	87.2	0.757	0.89
CAM	78.0	80.0	73.9	86.4	64.2	0.831	0.80
Dysplasia	76.6	75.0	77.2	56.6	88.6	0.645	0.80
All abnormalities	71.6	67.3	85.3	93.5	45.3	0.783	0.81

**Figure 1.**
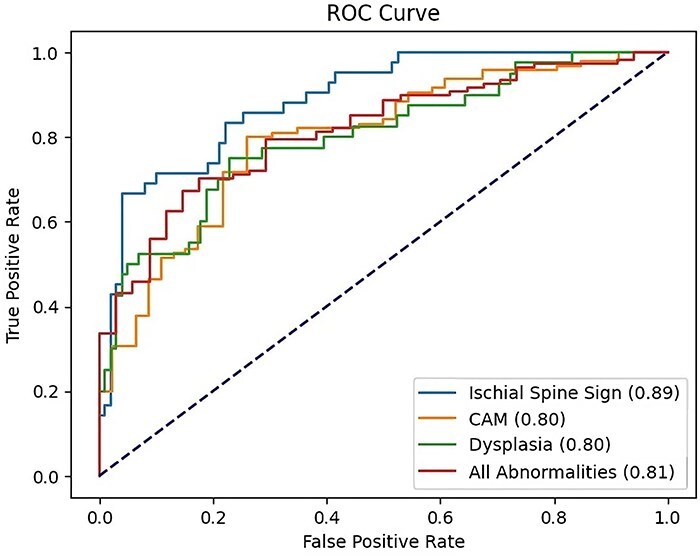
ROC curves for the DL model in detecting various hip joint abnormalities.

The receiver operating characteristic (ROC) curves for each abnormality, as shown in Fig. 1, illustrate the model’s strong performance. The model achieved an area under ROC (AUROC) of 0.89 for ischial spine sign, 0.80 for cam deformity, 0.80 for dysplasia, and 0.81 for all abnormalities, demonstrating the model’s ability to distinguish between the presence and absence of these characteristics. As sex was an auxiliary objective that was easy for the model to predict, it achieved an accuracy of 1.00 and an AUROC of 1.00 in predicting patients’ sex.

Inter-rater agreement, assessed using Gwet’s AC1, was substantial for dysplasia (0.83) and all abnormalities (0.88), and moderate for ischial spine sign (0.75) and cam deformity (0.61).

**Figure 2.**
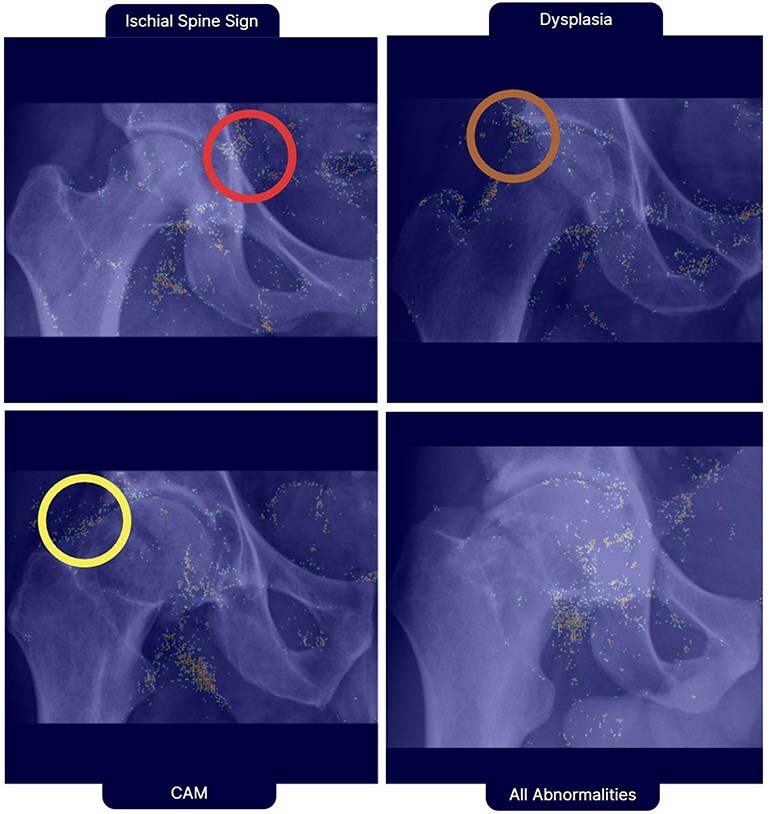
Grad-CAM visualizations highlighting the ROIs that the DL model focuses on detecting ischial spine sign, dysplasia, cam deformity, and all abnormalities combined. The visualizations provide insights into the model’s decision-making process and the ability to identify relevant anatomical landmarks for each abnormality. The model accurately localizes the ischial spine (red circle) for detecting the ischial spine sign, the lateral acetabular edge (orange circle) for dysplasia, the femoral head–neck junction (yellow circle) for cam deformity, and all relevant regions for detecting any abnormalities.

Grad-CAM visualizations (Fig. 2) highlight the ROIs that the model focuses on when making predictions. These visualizations provide valuable insights into the model’s decision-making process and demonstrate its ability to identify relevant anatomical landmarks for each abnormality.

## Discussion

The current study introduces a novel approach utilizing DL techniques for the automated detection of morphological hip pathologies, focusing on DDH and FAI. The results demonstrate the efficacy of a ConvNeXt-Tiny model trained on a dataset of AP pelvic radiographs, achieving good, but nowhere near perfect, accuracy in predicting various hip joint characteristics, including ischial spine sign, cam deformity, dysplasia, and other abnormalities. Expert inter-rater reliability was also good, but not perfect. These results show the promise and current limitations of using plain radiographs for simple morphological hip classifications, and the downstream impact this has on developing reliable DL models.

The current study’s model contributes to the growing body of evidence supporting the application of DL in musculoskeletal radiographic analysis. Previous studies successfully utilized DL methods for diagnosing hip osteoarthritis, osteoporosis, sacroiliitis, avascular necrosis, and other hip pathologies based on pelvic radiographs [19–23]. Notably, the study extends this paradigm to the diagnosis of FAI and DDH, demonstrating the feasibility of using DL algorithms to identify morphological abnormalities indicative of over or under-coverage of the femoral head by the acetabulum and bony abnormalities within the femur and pelvis.

FAI and DDH present a significant challenge in clinical practice, often leading to secondary hip osteoarthritis and debilitating symptoms in young individuals. While advanced imaging modalities like computer tomography (CT) and magnetic resonance imaging (MRI) offer detailed assessment of hip joint morphology, conventional radiography remains the initial imaging modality due to its accessibility and ease of evaluation. The accurate interpretation of radiographic parameters can be challenging, particularly in borderline cases, leading to misdiagnosis and delayed treatment. DL algorithms can aid in the precise measurement and interpretation of radiographic parameters, improving diagnostic accuracy and reducing inter-observer variability. Furthermore, integrating artificial intelligence (AI) into radiology information systems and picture archiving and communication systems can streamline the diagnostic workflow by enabling automatic identification and classification of findings, generating recommendations, and facilitating surveillance and research studies.

Compared to previous studies, the current study demonstrates promising advancements in the automated detection of morphological hip pathologies using DL techniques. Hoy *et al*. achieved a convolutional neural network (CNN) accuracy of 74% for identifying cam-type FAI morphology with high sensitivity (82%) but comparably low specificity (67%) [7]. Atalar *et al*. reported an accuracy of 87% using a pretrained VGG-16 model for FAI diagnosis with impressive performance metrics including 83% sensitivity, 90% specificity, 86% precision, 0.84 F1 score, and 0.92 AUC [8]. Xu *et al*.’s AI-aided diagnostic system used a mask-region-based CNN object detection model followed by a high-resolution network for landmark detection/extraction and a ResNet50 model for classification, showing accuracies ranging from 86% to 95% in providing appropriate Tonnis and International Hip Dysplasia Institute (IHDI) classifications. This system also showed intraclass consistency of acetabular index and center-edge angle among surgeons of 0.79–0.98, while AI showed perfect agreement. Additionally, the algorithm took significantly less time than a group of surgeons to draw conclusions [10]. Fraiwan *et al*. employed a deep transfer learning technique using a DarkNet53 model that achieved very high accuracy at 96% and perfect sensitivity but with a comparably lower specificity of 94% [9]. Liu *et al*. employed a pyramid nonlocal U-Net model to measure Tonnis angle with 90% accuracy [11]. Al-Bashir used Canny edge detection to identify pertinent radiographic features followed by bread first search to identify center of the femoral head. It then applied Hough transform for edge detection to find center edge angle and Tonnis angle with accuracies ranging from 46% to 78% and 84% to 85%, respectively [24]. In comparison, the current study examined a cohort of 500 patients, demonstrating robust performance in detecting various hip joint abnormalities from AP pelvic radiographs. (Table 2)

**Table 2.** Performance of various models in detecting morphological features on radiographs.

Morphologic feature	Study	Accuracy (%)	Sensitivity (%)	Specificity (%)	PPV (%)	AUC	AUROC	F1 Score
Cam deformity [7]	Hoy *et al*.	74	82	70	–	0.74	–	–
	Current study	78	80	74	86	–	0.80	0.83
Ischial spine sign	Current study	87	67	96	88	–	0.89	0.76
FAI diagnosis [8]	Atalar *et al*.	87	83	90	86	0.92	–	0.84
Dysplasia/DDH [9]	Fraiwan *et al*.	96	100	94	91	–	–	95%
	Current study	77	75	77	57	–	0.80	0.65
Tonnis angle [11, 24]	Liu *et al*.	90	–	–	–	–	–	–
	Al-Bashir *et al*.	84–85	–	–	–	–	–	–
Center-edge angle [24]	Al-Bashir *et al*.	46–78	–	–	–	–	–	–
Shenton’s line [10]	Xu *et al*.	92–95	92–96	88–91	–	–	–	–
Lateral edge of acetabulum [10, 11]	Xu *et al*.	89–90	87–89	93	–	–	–	–
	Liu *et al*.	90			90			
Sourcil [10]	Xu *et al*.	86–87	84–85	88–90	–	–	–	–
Any abnormality	Current study	72	67	85	94	–	0.81	0.78

The current DL model achieved high accuracy in detecting cam deformity of 78.0% with notable sensitivity and specificity values of 80.0% and 73.9%, respectively. Additionally, the model’s performance in identifying ischial spine sign showed commendable accuracy and specificity of 87.2% and 96.0%, respectively, but with comparably lower sensitivity of 66.7%. Detection of dysplasia exhibited good accuracy of 76.6%, sensitivity of 75.0%, and specificity of 77.2%. Detection accuracy of all abnormalities was 71.6%, with a lower sensitivity of 67.3% and higher specificity of 85.3%. These results highlight the model’s capability to accurately distinguish between the presence and absence of various hip joint characteristics.

Moreover, the performance metrics further demonstrate the model’s efficacy, achieving AUROCs of 0.89 for ischial spine sign, 0.80 for cam deformity, 0.80 for dysplasia, and 0.81 for all abnormalities combined. These high AUROC values underscore the model’s moderate ability to identify hip pathologies from radiographic images.

Inter-reader agreement was calculated using Gwet’s AC1, a metric resistant to the prevalence effect and the assumption of independence between observers—limitations commonly associated with the Kappa statistic [25]. Gwet’s AC1 is suitable for the current study, in which the balanced assessment of agreement among annotators is crucial, regardless of the class distribution or the independence of observations. Assessment of inter-rater agreement using Gwet’s AC1 revealed substantial agreement for dysplasia (0.83) and all abnormalities (0.88), and moderate agreement for ischial spine sign (0.75) and cam deformity (0.61). These results indicate the limitations in achieving consensus among human evaluators, which creates challenges in creating ground truth data in any hip preservation study based on radiographs alone (especially if angles are not utilized in classification) and, more importantly to the current study, precludes a clean ground truth for comparison by the DL model.

Grad-CAM visualizations provide valuable insights into the ROIs that the model focuses on when making predictions, highlighting its ability to identify relevant anatomical landmarks for each abnormality. When evaluating for CAM deformity, ischial spine sign, and dysplasia, the model notes ROIs along the lateral and medial aspect of the femoral head, the area medial to the iliopectineal line, and the anterolateral aspect of the acetabulum, respectively.

It is important to highlight the simplicity of the current study’s approach. The model examined solely the presence of two signs of FAI (ischial spine sign, cam deformity) or dysplasia rather than conducting a comprehensive workup involving complex landmark detection and feature extraction to measure continuous metrics such as Tonnis angle, center-edge angles, and extrusion index. Clinicians often utilize 3D imaging modalities such as MRI and CT scans as the standard of care for morphological hip classification due to their ability to provide detailed assessments. However, the current paper deliberately examined the most basic form of hip pain workup—radiographs without accounting for angles—to evaluate whether DL could effectively classify simple measures with performance comparable to human interpretation. As such, this study serves as a proof-of-concept before incorporating more comprehensive metrics in subsequent works.

The study’s limitations include the retrospective design, which restricted the evaluation to AP radiographs without angle measurements, potentially overlooking cases where additional imaging modalities may provide supplementary diagnostic information. In future research endeavors, it would be advantageous to expand the analysis to continuous variables as well as to incorporate 3D imaging modalities such as MRI and CT scans. These additions will provide a more comprehensive understanding of hip morphology and further validate the applicability of DL techniques in musculoskeletal radiographic interpretation.

The current study highlights the potential and inherent limitations of DL methods in augmenting diagnostic capabilities for hip pathologies, particularly FAI and DDH, through automated analysis of morphology present on pelvic radiographs. While further validation and refinement are necessary, the findings suggest a promising avenue for leveraging AI-driven technologies to enhance musculoskeletal radiographic interpretation and improve patient care. However, they also introduce a strong note of caution in training models based on metrics upon which even experts have poor consensus that will influence ultimate model capability.

## Data Availability

The data underlying this article are available upon reasonable request from the corresponding author.
